# Real time MRI pulse wave velocity for exercise stress testing

**DOI:** 10.1186/1532-429X-14-S1-P228

**Published:** 2012-02-01

**Authors:** Paul Roberts, Brett R Cowan, Alistair A Young

**Affiliations:** 1 Auckland Bioengineering Institute, University of Auckland, Auckland, New Zealand; 2 Faculty of Medical and Health Sciences, University of Auckland, Auckland, New Zealand

## Background

Pulse wave velocity (PWV) has been established as an independent predictor of CVD and has been shown to increase with age and pathology. PWV measurement during exercise stress testing may provide useful information beyond that seen in the resting state on the progression of disease. We sought to develop a system and protocol for the measurement of PWV and LV function in exercise.

## Methods

LV function was assessed by reduced-slice guide-point modelling from 6 SA slices and 2 LA slices acquired using a tGRAPPA accelerated SSFP sequence, as previously validated [1]. Two slices were acquired per breath-hold with a breath-hold duration of 5-8 sec. PWV was acquired in real time at 7.8 msec temporal resolution between two slices located at the pulmonary bifurcation and just above the renal and superior mesenteric arteries, using a RACE flow sensitive sequence [2]. Phase maps were processed to remove stationary tissue signal [2] and flow velocity extracted from the selected region of interest. PWV was estimated from the foot to foot time of the velocity wave and distance between slices. PWV estimation was validated in a flexible hose phantom with a pulsatile flow pump against pressure recordings. Exercise within the scanner was achieved with a custom-built MRI compatible cycle ergometer fixed to the patient table. 19 healthy volunteers age 22 to 73 years (11 male) exercised to a work rate of 34±11 W in order to increase their resting heart rate by ~30 bpm. Motion was suspended for each breath-hold acquisition. This was repeated four times for complete LV function, and two times for PWV capturing 10-20 beats. PWV was calculated for each beat and averaged. Central pressures were estimated using a PulseCor brachial cuff pressure system.

## Results

**Figure 1:**
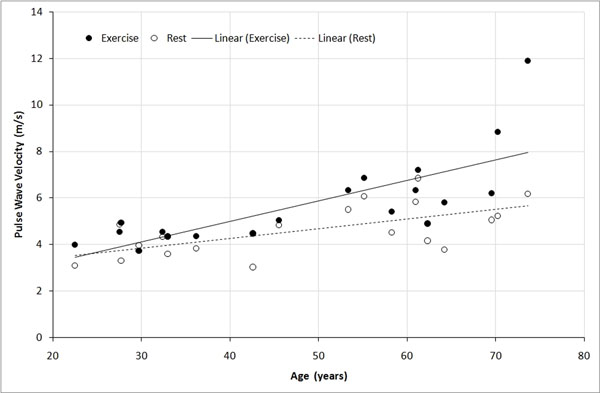
PWV at rest and after exercise in healthy volunteers.

PWV in the pulsatile flow phantom was 22 m/s by pressure wave and 20 m/s by MRI RACE. Table 1 shows LV function, central pressure and aortic PWV during rest and exercise for the healthy volunteers. Figure 1 shows PWV with respect to age at rest and immediately after moderate exercise. The results are similar to those previously reported using longer gated MRI acquisitions [2,3]. PWV was correlated with central systolic pressure in both rest and exercise (r=0.46 and r=0.36 respectively). In young subjects there was negligible difference between rest and exercise PWV, however with increased age a difference became evident (p=0.033), even after normalizing for central pressure.

**Table 1:** PWV and LV function at rest and after exercise

		Rest	Exercise	p-value*
Pulse Wave Velocity	m/s	4.6±1.1	5.8±2.0	<0.01
Systolic BP	mmHg	117±15	131±17	<0.001
Diastolic BP	mmHg	73±13	75±11	0.052
Central Systolic BP	mmHg	108±15	119±16	<0.001
Stroke Volume	mL	92±15	104±21	<0.001
Ejection Fraction	%	63±3	69±3	<0.001
Cardiac Output	L/min	6.1±1.2	10.0±1.7	<0.001
Heart Rate	bpm	68±11	97±10	<0.001
Work Rate	W	-	34±11	-

* paired t-test

## Conclusions

PWV and LV function during exercise stress testing can be performed in just six breath-hold acquisitions of 5-8 sec duration. Both central pressure and PWV were elevated in exercise, with greater elevations in older subjects.

## Funding

Project funding was provided by Health Research Council of New Zealand and the National Heart Foundation of New Zealand.
